# Effect of Different Surface Designs on the Rotational Resistance and Stability of Orthodontic Miniscrews: A Three-Dimensional Finite Element Study

**DOI:** 10.3390/s21061964

**Published:** 2021-03-11

**Authors:** Jin-Young Choi, Jaehee Cho, Song Hee Oh, Seong-Hun Kim, Kyu-Rhim Chung, Gerald Nelson

**Affiliations:** 1 Department of Orthodontics, Graduate School of Dentistry, Kyung Hee University, Seoul 02447, Korea; joyful.ortho@gmail.com (J.-Y.C.); speedor@hotmail.com (K.-R.C.); 2 Department of Orthodontics, Graduate School of Medicine, Korea University, Seoul 02841, Korea; jhcho.seoultop@gmail.com; 3 Department of Oral and Maxillofacial Radiology, Graduate School of Dentistry, Kyung Hee University, Seoul 02447, Korea; ohbbang50@gmail.com; 4 Division of Orthodontics, Department of Orofacial Science, University of California, San Francisco, CA 94143, USA; gdnelson41@gmail.com

**Keywords:** implant, macrostructural modification, osseointegration, surface treatment

## Abstract

High orthodontic forces and various directions of applied forces can be associated with loosening of the screw anchorage in the bone. Screw designs have been modified to increase the stability of the miniscrews. This research evaluates the influence of three-designs on the stability of orthodontic miniscrews. A conventionally cylinder-type miniscrew design (Bio-Action screw, Jin-Biomed co., Bucheon, Korea) was set as a control, and three conditions were studied based on modifications of this control design. Condition-1 has narrowed threads in the upper part of the screw; Condition-2 has a notch at the middle part; and Condition-3 has the combination of Condition-1 and Condition-2. The moment required to unwind the miniscrew to five degrees is tested, and the moment generated at the cortical bone and the trabecular bone were calculated with finite element analysis. Compared to the control, all three conditions showed a higher moment required to unwind the miniscrew and a higher moment generated at the cortical bone. At the trabecular bone, condition-2 and -3 showed higher moment than the control, and condition-1 showed similar moment to the control. Condition-3 required a higher overall moment to unwind the miniscrew. These findings validate the design modifications used to increase the rotational resistance.

## 1. Introduction

In contemporary orthodontics, mechanical design enhancements have improved the development of orthodontic materials. One of the greatest advances affecting the transition to contemporary orthodontics is the introduction of orthodontic miniscrews. Orthodontic miniscrews have been used for bodily retraction of anterior teeth [1], intrusion of incisors for deep bite correction [2], intrusion of molars in patients with anterior open bite [3], uprighting of impacted teeth [4], and other applications. Multiple orthodontic miniscrews have been used to assist lateral expansion of the palate in patients with a constricted palate that results in a transverse discrepancy between the maxilla and mandible [5]. Stability rates or failure rates of orthodontic miniscrews have been studied. Both metrics are important to clinicians. In a meta-analysis study of the failure rates and associated risk factors of orthodontic miniscrews, the overall failure rate was 13.5%. The failures were associated with the choice of jaw insertion, but not with the sex or age of the patient or side of miniscrew insertion [6]. Orthodontic miniscrews and dental implants share similarity in that they both are implanted into alveolar bone consisting of a cortical outer layer and a trabecular inner layer. The distinctive and critical feature of dental implants is that they are designed to integrate with the bone to achieve long term stabilization, which is called as osseointegration [7,8,9]. Researchers have tried to improve bone-to-implant contact by modification of the surface characteristics of dental implants. For example, the surface of the titanium implant was first sandblasted and then acid-etched, and it was called as sandblasted, large grit, acid-etched (SLA) treatment [10,11]. It enhanced the surface roughness to improve the osseointegration. A study using high-resolution transmission electron microscopy (HRTEM), energy dispersive X-ray spectroscopy (EDS), and scanning TEM (STEM)/electron energy loss spectroscopic analysis (EELS) confirmed the nanoscale osseointegration and the possibility of the existence of osseohybridization zones between the bone and implant surface [12]. When orthodontic miniscrews were first developed, the presence and the necessity of osseointegration was a controversy. Orthodontic implants had a smaller diameter than dental implants, which allowed new placement sites, such as the inter-radicular space [13,14], the supra-apical and infra-zygomatic area [14,15,16,17], and the mandibular symphyseal area [14]. In those areas, some authors insisted that the retention of the miniscrew and titanium pin had been achieved only by a mechanical fixation, not by the osseointegration [18]. However, the consensus of recent studies is that partial osseointegration of the orthodontic miniscrews is necessary, even if they are used as a temporary skeletal anchorage device, because any encapsulation of connective tissue leads to the loosening of the miniscrew or mini-implant [19,20,21]. In addition, clinicians want to expand the variety of force applications to include heavier, dynamic, and rotational forces especially for biocreative orthodontic strategy treatment [12,22]. When the orthodontic miniscrew is placed into the alveolar bone, it is inserted in the clockwise rotational direction. Clinically applying a counterclockwise rotational force may tend to unscrew and loosen the miniscrew so that increased rotational resistance of the orthodontic miniscrew is required.

Previous studies have focused on the microstructural modification of the dental implant or the orthodontic miniscrew surfaces to facilitate the osseointegration between the surface of dental implant or orthodontic miniscrew and bone [23,24]. Recently, in addition to treating surface of the orthodontic miniscrew such as SLA treatment, ozone therapy and photobiomodulation were introduced for a better osseointegration process [25]. Another attempt to stimulate peri-implant osteogenesis by ultraviolet photofunctionalization was tried, but it did not enhance the biologic potential of orthodontic miniscrew [26]. By changing the point of view, we hypothesized that a macroporous condition through a geometric change of the orthodontic miniscrew surface would provide similar rotational resistance to the osseointegrated microporous condition. The aim of this study was to investigate the rotational resistance of the orthodontic miniscrew with modifications of the surface at the macrostructural level, using finite element analysis.

## 2. Materials and Methods

### 2.1. Development of Finite Element Model

**Figure 1:**
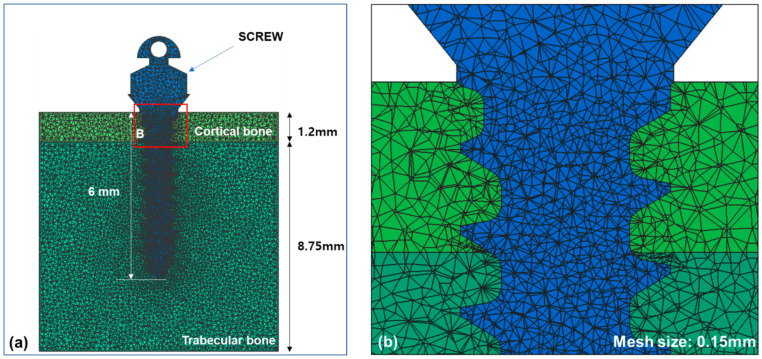
Finite element models for the alveolar bone and an orthodontic miniscrew were constructed: (**a**) An orthodontic miniscrew was implanted into the alveolar bone in the finite element model. Total length of the thread part of the orthodontic miniscrew was set at 6 mm and the top of the thread was completely placed into cortical bone; (**b**) A magnified image of the contacted interface of bone and the surface of orthodontic miniscrew. The condition of contact was set up as surface to surface contact with an algorithm of the argumented Lagrangian method and the coefficient of friction was set at 0.3.

To construct a finite element model with tetrahedral meshes, the information of alveolar bone with cortical and trabecular bone was imported into Visual-mesh software (version 7.0; ESI group, Paris, France). The thickness of cortical bone was set at 1.2 mm and the trabecular bone underneath was deep enough to place an orthodontic miniscrew with thread length of 6.0 mm. The information of an orthodontic miniscrew was also imported and converted to a finite element model. Based on the previous research [22,23,24,25,26,27,28], we modified a C-implant and used it as an orthodontic miniscrew. A diameter of the orthodontic miniscrew we used was 1.6 mm, smaller than that of the original C-implant at 1.8 mm (Bio-Action screw (BA screw), Jin-Biomed co., Bucheon, Korea). The orthodontic miniscrew was designed as a cylinder-type, which was thickest on the top part of the thread and thinner at the tip. The material is Ti-Grade-V-alloy (Ti-6Al-4V, body centered cubic structure) which is an alloy combined α-β phases [29], and has cutting flutes at the apex. The orthodontic miniscrew was placed into the alveolar bone model completely so that the top part of the thread was surrounded by the cortical bone. The condition of contact was set up as surface to surface contact with an algorithm of the augmented Lagrangian method, and the coefficient of friction was set at 0.3. The mesh size of the finite element model was set at 0.15 mm (Figure 1). The material properties of each component are shown in Table 1. All the components were considered to be homogenous and isotropic.

### 2.2. Modification of Miniscrew Design

**Figure 2:**
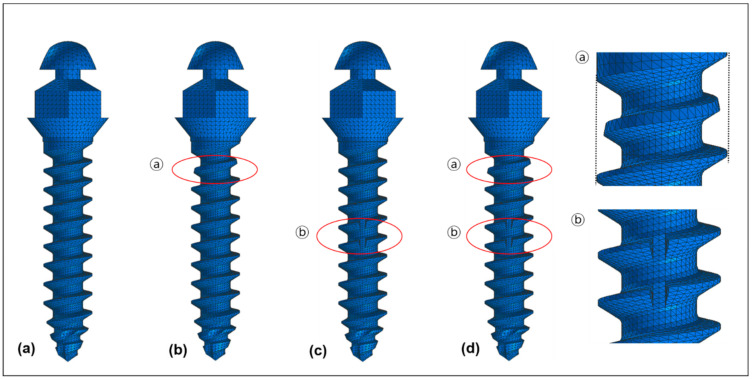
Four models of orthodontic miniscrews used in the finite element analysis: (**a**) A control showing the original cylinder type of threads with 1.6 mm of diameter at the upper part and 1.5 mm of diameter at the apex part; (**b**) Condition 1 showing the narrowed thread (ⓐ) at the uppermost part of the threads; (**c**) Condition 2 showing the vertical notch (ⓑ) at the middle of the threads; (**d**) Condition 3 showing the combination of two modifications (ⓐ + ⓑ).

The original design of the orthodontic miniscrew was used as a control (Figure 2a). It is a cylinder type miniscrew with 1.6 mm of diameter at the upper (neck) part and 1.5 mm of diameter at the apex part. The threads narrow evenly going toward the apex. Two modifications were applied according to our hypotheses. The first modification was a narrowed thread at the uppermost part of miniscrew. The second modification was a vertical notch at the middle part of the miniscrew. Based on the combination of the modifications, three models were set up. Condition 1 had only the first modification (Figure 2b), and condition 2 had only the second modification (Figure 2c), while condition 3 had both modifications (Figure 2d).

### 2.3. Force Loading Conditions

**Figure 3:**
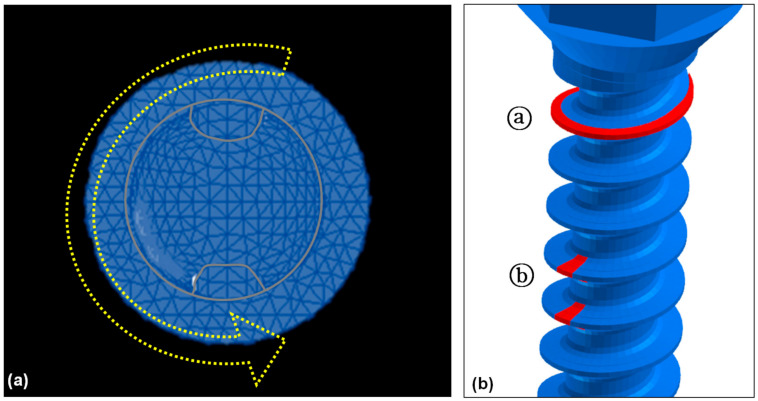
Force loading conditions to measure the rotational resistance of the orthodontic miniscrew: (**a**) Top view of the orthodontic miniscrew with a hole on the head. Yellow arrow indicates the counterclockwise rotating direction to unwind the orthodontic miniscrew; (**b**) Detailed view of two modifications in the threads of the orthodontic miniscrew. Red-colored part of the threads were cut additionally for the increase of the resistance. The uppermost part of the threads was narrowed (ⓐ), and the vertical notch was added at the middle of the threads at the uppermost part of the threads (ⓑ).

Insertion of the miniscrew is with a clockwise rotation. Force was applied in a counterclockwise rotating direction to measure the rotational resistance (Figure 3). All four models were rotated for 5 degrees. Von-Mises stress at the upper third, the middle third, and the apical third of the orthodontic miniscrew, and the overall Von-Mises stress were measured under 5 degrees of counter-clockwise rotation of the control and the orthodontic miniscrews with each modification. In addition, the moment generated on the miniscrews was calculated at every 1 degree. The overall moment, moment at the middle of cortical bone, and moment at the middle of trabecular bone were measured in each condition. The force loading simulations were performed by a Virtual Performance Solution (version 2008; ESI group, Paris, France) on a computer with Intel^®^ Xeon^®^ CPU E5-2680 @ 2.40 GHz X 28 core and the 128 GB RAM.

### 2.4. Statistical Analysis

We performed a generalized linear regression analysis to figure out the association between resisting moment and counterclockwise rotation of the orthodontic miniscrew for control and each condition. In addition, we analyze the significance of differences among the control and condition 1 to 3 through Bonferroni’s post-hoc analysis. Statistical analyses were performed using SAS 9.4 program (SAS Institute Inc., Cary, NC, USA), and the significant value was 0.05.

## 3. Results

### 3.1. Von-Mises Stress

#### 3.1.1. Bone versus Orthodontic Miniscrew

Under 3 conditions and the control, the maximum Von-Mises stress was higher at the orthodontic miniscrew than at the bone. The differences were smallest in the control suggesting 1.6-fold higher at the orthodontic miniscrew (0.045 GPa at the bone and 0.072 GPa at the orthodontic miniscrew), and largest in the condition 2 showing about 7.6-fold higher at the orthodontic miniscrew than at the bone (0.034 GPa at the bone and 0.260 GPa at the orthodontic miniscrew) (Table 2).

#### 3.1.2. Area of the Orthodontic Miniscrew

**Figure 4:**
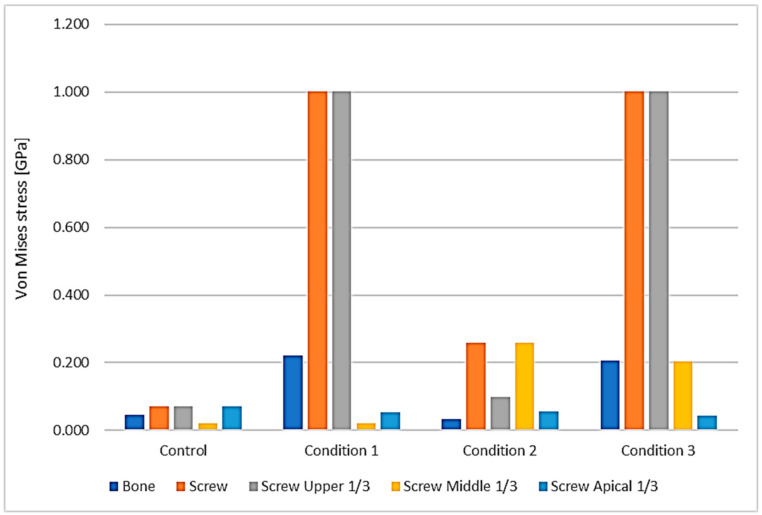
Distribution of the maximum Von-Mises stress at the bone and at the orthodontic miniscrew. The area of the miniscrew was divided into three parts according to their vertical positions: the upper third (gray), the middle third (yellow), and the apical third (sky blue). Maximum Von-Mises stress of each part is visualized in the graph. The orange one is the maximum Von-Mises stress of all screw parts. Upper third of the screw part showed the maximum Von-Mises stress in condition 1 and 3, while the middle third of the screw part showed the maximum Von-Mises stress in condition 2. In all the conditions, the maximum Von-Mises stresses of bone and entire screw were much higher than the control, especially in conditions 1 and 3.

**Figure 5:**
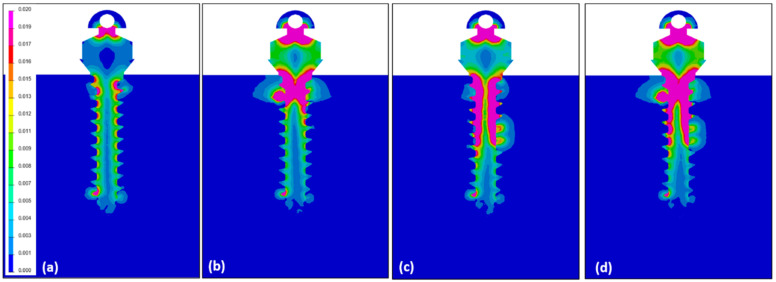
Illustrations showing the distribution of Von-Mises stress under counter-clockwise loading of 5° of the miniscrew: (**a**) In the control, the Von-Mises stress was distributed evenly and the amount of the stress was low; (**b**) In condition 1, a high Von-Mises stress was concentrated at the upper third of the orthodontic miniscrew; (**c**) condition 2 had a relatively even stress distribution, but the value was low as seen in Figure 4 and Table 2; (**d**) In condition 3, the stress was mostly concentrated at the upper third as in condition 1, but a quite high stress was focused at the middle notched area.

Depending on the surface modification of the miniscrew, the focused area of maximum Von-Moses stress differs. In the control miniscrew, the upper third and the apical third both were the area of highest stress, 0.072 GPa. In the orthodontic miniscrews with condition 1 and condition 3, the highest Von-Mises stress was at the upper third of the orthodontic miniscrew, recording 1.002 GPa and 1.001 GPa, respectively. On the other hand, the orthodontic miniscrew with condition 2 with a vertical notch, had the highest Von-Mises stress, 0.260 GPa, at the middle third, where the vertical notch was present (Figure 4). These results were also confirmed in Figure 5. In the control, the stress was distributed evenly and the value of Von-Mises stress was low. In condition 1, the stress was concentrated at the upper third, while the condition 2 showed relatively even distribution of the stress. In condition 3, the stress was concentrated at the upper third as in condition 1. However, in condition 3, the Von-Mises stress at the middle third was much higher than the that in condition 1.

#### 3.1.3. Modification of the Surface of the Orthodontic Miniscrew

With the conventional miniscrew of the cylindrical type without any modification (our control example), the stress was distributed relatively evenly on the orthodontic miniscrew, with only 0.072 GPa of maximum Von-Mises stress over all parts. However, in all three modified surface conditions the maximum Von-Mises stress was much higher than the control. The model with a vertical notch at the middle third of the orthodontic miniscrew (condition 2) showed 0.260 GPa of maximum Von-Mises stress which was about 3.6 times higher than the control. In addition, models with a narrowed uppermost thread of the orthodontic miniscrew (conditions 1 and 3), regardless of the presence of the vertical notch at the middle third of the orthodontic miniscrew, indicated 1.001 to 1.002 GPa of maximum Von-Mises stress which was about 13.9 times higher than the control.

### 3.2. Moment Required to Unwind the Orthodontic Miniscrew

#### 3.2.1. Overall Moment

**Figure 6:**
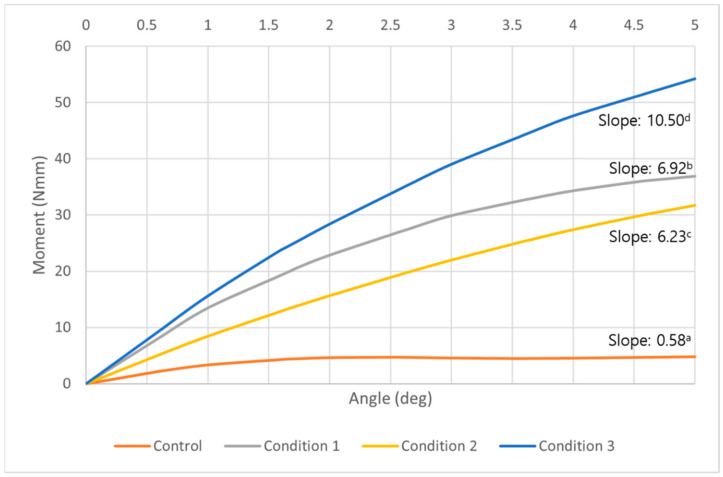
A graph of the overall moment required to unwind the orthodontic miniscrew at each degree of counterclockwise rotation in control (orange), condition 1 (gray), condition 2 (yellow), and condition 3 (blue). While the models of all conditions showed a constant increase of the moment, the control showed an increase by 2 degrees of counterclockwise rotation, but a plateau appeared from 2 degrees to 5 degrees of the counterclockwise rotation. The highest moments were measured at every angle in condition 3 model. ^a,b,c,d^ Different alphabets means statistically different slopes in control and each condition (*p* < 0.05).

The overall moment generated on the entire miniscrew increased in all three conditions. The control had an increase of moment by about two degrees of counterclockwise rotation, but from 2 to 5 degrees the increase plateaued. At five degrees of counterclockwise rotation, the condition 3 example had the highest moment (Figure 6). The moment required to unwind the miniscrew in condition 3 was about 11.3 times higher than the control. At the same degrees of counterclockwise rotation, condition 1 and condition 2 showed about 7.7 times and 6.6 times higher moment than the control, respectively. The control and all conditions showed statistically significant increases as the angle of counterclockwise rotation increased, and the differences among the control and each condition were also significant (*p* < 0.05) (Table 3).

#### 3.2.2. Moment at the Cortical Bone

**Figure 7:**
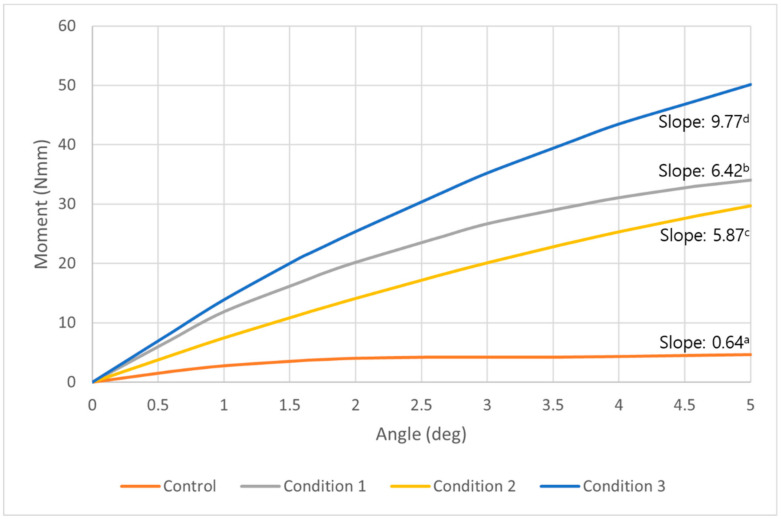
A graph of the moment at the middle of cortical bone required to unwind the orthodontic miniscrew in control (orange), condition 1 (gray), condition 2 (yellow), and condition 3 (blue). This graph shows a tendency similar to the graph of the overall moment in Figure 6. Three conditions all show constant increases as the degrees were raised to 5 degrees in counterclockwise rotational direction. The condition which showed the most increased moment was condition 3, followed by condition 1 and 2. ^a,b,c,d^ Different alphabets means statistically different slopes in control and each condition (*p* < 0.05).

At the cortical bone level, a similar tendency of moment change observed as overall moment increase. All three conditions showed a constant increase of the moment with progressing degrees of counterclockwise rotation. While the control showed a plateau of increase after about two degrees of counterclockwise rotation (Figure 7). Compared to the control, a moment 7.3, 6.4, and 10.8 times higher was required to unwind the miniscrew in condition 1, 2, and 3, respectively. The control and all conditions showed statistically significant increases as the angle of counterclockwise rotation increased, and the differences among the control and each condition were also significant (*p* < 0.05) (Table 4).

#### 3.2.3. Moment at the Trabecular Bone

**Figure 8:**
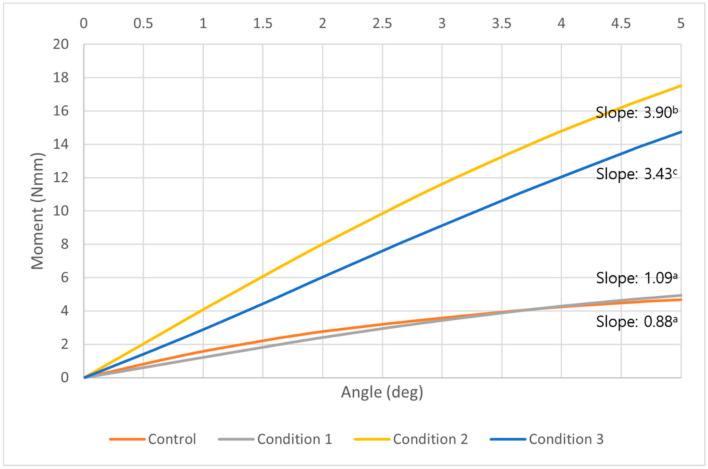
A graph of the moment at the middle of trabecular bone required to unwind the orthodontic miniscrew in control (orange), condition 1 (gray), condition 2 (yellow), and condition 3 (blue). At all degrees, the model with condition 2 showed the highest moment than any other conditions or the control. A model with condition 1, with the narrowed thread diameter at the upper part, showed a similar moment pattern to the control. All models including the control showed a constant increase as the angle of counterclockwise rotation increased. ^a,b,c^ Different alphabets means statistically different slopes in control and each condition (*p* < 0.05).

In the middle of the trabecular bone, all models including the control showed constant increase along with the increase of counterclockwise rotational degrees. We found that the model of condition 2 had the highest moment among all conditions, unlike the results of the overall moment or in the cortical bone. In addition, the control model and condition 1 model showed a similar pattern of moment changes (Figure 8). At five degrees of counterclockwise rotation, the moment of condition 2 was 4.2 times higher than the control, and the condition 3 was 3.6 times higher moment than the control. The control and all conditions showed statistically significant increases as the angle of counterclockwise rotation increased. The differences among the control and each condition were also significant (*p* < 0.05) except between the control and condition 1 (Table 5).

## 4. Discussion

This study demonstrates that the combination of narrowing the uppermost thread and placing a vertical notch at the middle part of the miniscrew will increase the rotational resistance without any surface treatment. A model with condition 1 showed a high moment to unwind the miniscrew as well as a model with condition 3. The Von-Mises stress was mostly concentrated at the upper third of the orthodontic miniscrew in condition 1. Condition 1 has some clinical disadvantages. Risk of fracture may be increased, and in a situation with thicker mucosa, the narrow thread area may not even reach the cortical bone. This second condition is common o the palatal side of the alveolus, where mucosal thickness has been measured at ~4 mm [30]. Clinicians have advised bi-cortical anchorage with the purpose of reduced overall cortical bone stress and good stability [31]. In a patient with sinus pneumatization bone support may not be possible at the apical end of the miniscrew. The main stability factor is rotational resistance at the cortical bone. It is known that the mechanical inter-digitation in the cortical bone is more critical to miniscrew stability than osseointegration at either the cortical or trabecular bone [32,33]. We count that condition 2, with a just the vertical notch at the middle part of the miniscrew, might not be enough to provide enough stability (Figure 6; Table 3). Consequently, the combination of both modifications, as with condition 3, offers the best choice for additional resistance to the unscrewing counterclockwise moment of the miniscrew.

**Figure 9:**
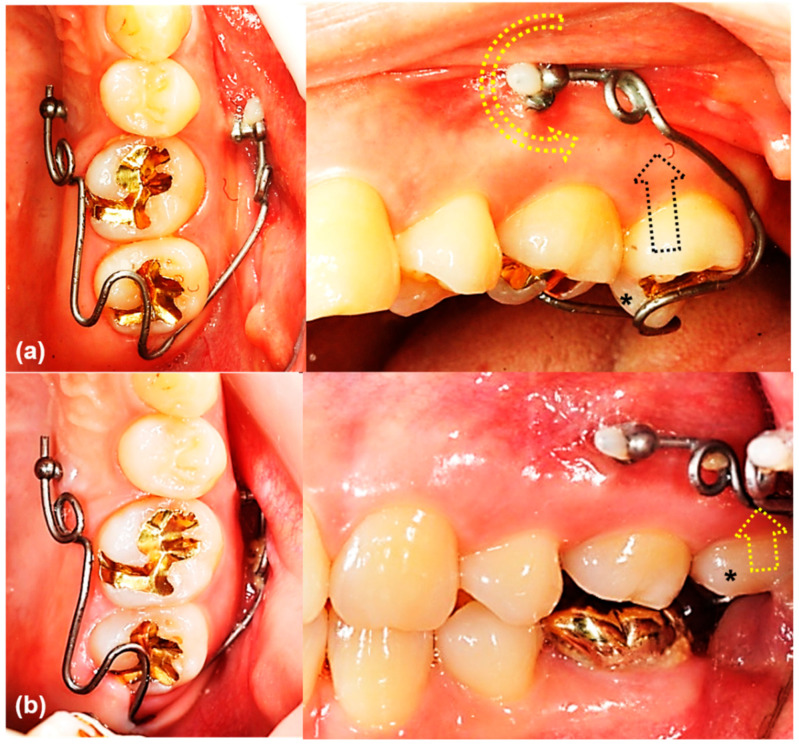
The orthodontic miniscrew supported the counterclockwise rotational force in a patient with extruded upper left second molar: (**a**) The patient has an extruded upper left second molar (*), especially the palatal cusp. To intrude this tooth, two orthodontic miniscrews (1.8 mm in diameter, 8.5 mm in length SLA surface treated mini-implant, C-implant, CIMPLANT co., Seoul, Korea) were inserted on the buccal and palatal sides between upper left second premolar and upper left first molar. A spring was designed to apply an intruding force (black dotted arrow) on the palatal cusp of the second molar, with its wire was passing through the holes of the orthodontic miniscrews. The spring generates a counterclockwise rotational moment on the buccal orthodontic miniscrew (yellow dotted arrow); (**b**) Six months later, the upper left second molar was intruded successfully. Both orthodontic miniscrews stayed stable in spite of the rotational moments.

**Figure 10:**
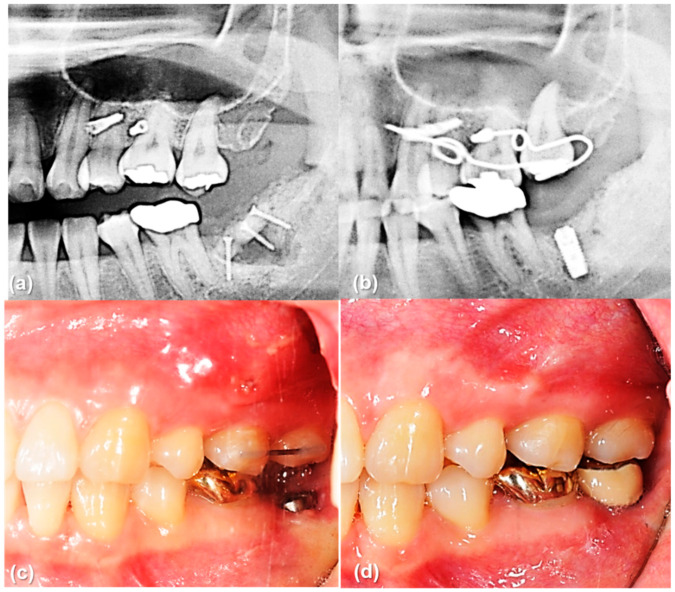
The orthodontic miniscrew supported the counterclockwise rotational force in a patient with extruded upper left second molar: (**a**) Pretreatment panoramic radiograph shows extrusion of upper left second molar. (**b**) Posttreatment panoramic radiograph shows sufficient intrusion of upper left second molar after six months of treatment. Dental implant was placed in the lower left second molar area. (**c**,**d**) Intraoral photographs before and after implant crown treatment.

The main result of this study that the rotational resistance of the orthodontic miniscrew with a novel design was increased would be applied to the clinical situation where the increased moment is required. Figure 9 and Figure 10 shows an example of the counterclockwise rotational force applied to an orthodontic miniscrew. A patient with an extruded upper left second molar was treated by one of the authors (K.R.C). The patient agreed and signed an informed consent form that the authors would use photos for the publication of an article. In this case, the orthodontic wire used to intrude the extruded upper left second molar was inserted into a hole in the heads of both orthodontic miniscrews between the upper left second premolar and the upper left first molar on the buccal and palatal side of alveolar bone. On the buccal side, the wire generated a counterclockwise rotational moment on the orthodontic miniscrew. If the amount of moment had been higher than the rotational resistance of the orthodontic miniscrew, the orthodontic miniscrew would have been loosened and the intrusion of the upper left second molar would have failed. In this case, the osseointegration was effective enough to stabilize the orthodontic miniscrew under the heavy moment of the counter-clockwise rotation.

Under loaded orthodontic forces on the orthodontic miniscrew, the Von-Mises stress is distributed differentially along the miniscrew and bone. Generally, the stress at the cortical bone is higher than the stress at the trabecular bone. In a three-dimensional finite element analysis with a bracket type orthodontic miniscrew, the maximum values of Von-Mises stress in the trabecular bone were much lower compared to the cortical bone. Only one hundredth of the stress in the cortical bone occurred in the trabecular bone [34]. Another finite element study with two different types of miniscrew suggested that the cortical bone absorbed most stresses, regardless of the type of miniscrew or the insertion angles [35]. In our study, a similar result could be inferred in that the upper third of the miniscrew, fitting to the cortical bone, had the maximum Von-Mises stress except in condition 2. However, in condition 3, the maximum Von-Mises stress was measured at the middle third. This correlates with the vertical notch of the thread. Influence of the vertical notch was critical to enhance the maximum stress at the middle third of the miniscrew even though it is the trabecular bone area.

In this study the moment required to unwind the miniscrew was assumed to be evaluated at least four weeks after placement. Berglundh et al. studied the alveolar bone formation adjacent to the implant at different phases of the osseointegration process [36]. New bone formation appeared at two weeks of healing, with a lining of bone-forming cells (osteoblasts) in the trabeculae of woven bone signaling the bone formation was initiated. After four weeks of implant installation, the newly formed bone was mineralized including woven bone combined with both parallel-fibered and lamellar bone. The effect of the modifications on the threads depends on new bone formation and mineralization inside the chambers between threads. In order to take advantage of the novel miniscrew design in the clinical situation, waiting for at least four weeks after implantation of miniscrew would be recommended for new bone formation at the vertical notch as well as between the pitches of the screw.

Removal of the orthodontic miniscrew is also important as it is used for only a few years during orthodontic treatment. So, we have called the orthodontic miniscrew as a temporary skeletal anchorage device. In some kinds of mini-implants placed on the palate, large amount of osseointegration similar to the dental implants is achieved in a few months, and the adjacent bone should be removed together with the mini-implant so that an extra healing period is required [18]. A 1.6 mm diameter of the partially osseointegrated orthodontic miniscrew which was modelled in this study is small enough to remove the miniscrew without any fracture or damage to the adjacent bone. In the study measuring the removal torque of the surface-treated orthodontic miniscrew with 1.8 mm of diameter, mean removal torque value (RTV) was 16.4 N per centimeter and the maximum RTV of all subjects was 35.41 N per centimeter [36]. Considering the fracture torque of common miniscrew with 1.6 mm of diameter is about 35 to 40 N per centimeter, the orthodontic miniscrew with a novel design used in this study would be expected to be safe during the removal procedure.

One of the surface-treated orthodontic miniscrews is the C-implant, with SLA treatment intended to enhance osseointegration [12,37,38]. The screw part of C-implant has a diameter of 1.8 mm. It is a smaller diameter than a dental implant for prosthesis, but 1.8 mm of diameter requires adequate space for placement in the interradicular space. Based on a recent systematic review with meta-analysis, most interradicular space is large enough to accommodate a 1.8 mm diameter miniscrew, but some interradicular space may be too small, as between the canine and the first premolar or between the first and second molars [39]. Considering this, we constructed the orthodontic screw model based on the C-implant, but modified its diameter from 1.8 mm to 1.6 mm. This smaller diameter has an advantage in decreasing the potential damage of adjacent roots, while guaranteeing a suitable stability.

The orientation of the miniscrew may also affect the stability. Pickard et al. measured the highest stability and resistance to failure when the forces were loaded in the same direction as the long axis of the miniscrew, while the least stability was found when the forces were loaded at an angle away from the long axis of the miniscrew, in their study with human cadaver mandibles [40]. They measured the failure from 87 to 342 N. We agree with the result that the highest resistance is achieved when the miniscrew is oriented perpendicular to the bone surface. So, we placed the miniscrew with an angle of 90 degrees to the bone surface in the virtual model. Of course, this is an experimental result, not based on a clinical situation. In the clinic, placing the orthodontic miniscrew at an angle to the bone surface may be inevitable to avoid the contact with other anatomic structures such as teeth roots. The orthodontic miniscrew we used in this study have a hole at its head part. The orthodontic wire inserted into this hole generates stresses and moments according to the implantation angle and the angle of force application, which concepts will benefit from further study.

The modifications used in this study are available for modifications of dental implants for prosthesis. Previous stability studies of dental prosthesis implants are focused on surface treatments. In addition to the microstructural surface treatment, the modifications of threads in a macrostructural scale would be helpful to maximize the rotational resistance of the dental implant. However, surface treatment of an orthodontic miniscrew with the two thread modifications described here would not be advised since the miniscrews are designed to be removed after orthodontic treatment.

## 5. Conclusions

We evaluated the effect of two orthodontic miniscrew thread modifications and their combination, narrowing the uppermost part of the thread and placing a vertical notch at the middle part of the miniscrew, to test the resistance against a counterclockwise rotation using a finite element analysis. Based on the results, two modifications of screw part were shown to be valid to increase the rotational resistance. Unlike previous orthodontic miniscrew which has large diameter (1.8 mm) and treated surface, the novel orthodontic miniscrew with new designs with its smaller diameter (1.6 mm) could serve without the need for microstructural surface-treatments. The concept of macrostructural surface modification, by extension, would be possibly applied to the dental implants.

## 6. Patents

Kyung Hee University Industry-University Cooperation Foundation own a patent of modified C-implant design (patent no. 10-2019-0102799).

## Figures and Tables

**Table 1:** The material properties used for finite element analysis.

	Young’s Modulus (GPa)	Poisson’s Ratio
Cortical bone	13.7	0.3
Trabecular bone	1.37	0.3
Titanium	110	0.35

**Table 2:** Maximum Von-Mises stress (GPa) at the bone and each part of the orthodontic miniscrew.

	Bone	Orthodontic Miniscrew			
		Overall	Upper 1/3	Middle 1/3	Apical 1/3
Control	0.045	0.072	0.072	0.020	0.072
Condition 1	0.221	1.002	1.002	0.022	0.054
Condition 2	0.034	0.260	0.099	0.260	0.056
Condition 3	0.206	1.001	1.001	0.204	0.045

**Table 3:** Overall moment (Nmm) required to unwind the miniscrew in each degree of counterclockwise rotation.

	Counterclockwise Rotation					Generalized Linear Regression *			
	1°	2°	3°	4°	5°	Slope	CI		*p*-Value
Control ^a^	3.34	4.62	4.55	4.53	4.78	0.58	0.35	0.81	<0.0001
Condition 1 ^b^	13.47	22.86	29.90	34.33	36.90	6.92	6.16	7.68	<0.0001
Condition 2 ^c^	8.46	15.66	21.99	27.40	31.73	6.23	5.94	6.25	<0.0001
Condition 3 ^d^	15.61	28.38	39.01	47.62	54.21	10.50	9.85	11.15	<0.0001

* It is an analysis to figure out effect of counterclockwise rotation to the moment. ^a,b,c,d^ Different alphabets showed significant differences analyzed by Bonferroni’s post-hoc test.

**Table 4:** Moment (Nmm) required to unwind the orthodontic miniscrew at the middle of the cortical bone in each degree of counterclockwise rotation.

	Counterclockwise Rotation					Generalized Linear Regression *			
	1°	2°	3°	4°	5°	Slope	CI		*p*-Value
Control ^a^	2.80	4.07	4.23	4.35	4.65	0.64	0.45	0.83	<0.0001
Condition 1 ^b^	11.88	20.22	26.72	31.12	34.08	6.42	5.81	7.04	<0.0001
Condition 2 ^c^	7.46	14.12	20.12	25.34	29.71	5.87	5.65	6.10	<0.0001
Condition 3 ^d^	13.90	25.41	35.30	43.60	50.31	9.77	9.26	10.29	<0.0001

* It is an analysis to figure out effect of counterclockwise rotation to the moment. ^a,b,c,d^ Different alphabets showed significant differences analyzed by Bonferroni’s post-hoc test.

**Table 5:** Moment (Nmm) required to unwind the orthodontic miniscrew at the middle of the trabecular bone in each degree of counterclockwise rotation.

	Counterclockwise Rotation					Generalized Linear Regression *			
	1°	2°	3°	4°	5°	Slope	CI		*p*-Value
Control ^a^	1.59	2.78	3.60	4.26	4.69	0.88	0.80	0.96	<0.0001
Condition 1 ^a^	1.31	2.58	3.69	4.65	5.45	1.09	1.05	1.13	<0.0001
Condition 2 ^b^	4.35	8.58	12.55	16.20	19.49	3.90	3.82	3.98	<0.0001
Condition 3 ^c^	3.18	6.65	10.15	13.59	16.89	3.43	3.41	3.45	<0.0001

* It is an analysis to figure out effect of counterclockwise rotation to the moment. ^a,b,c^ Different alphabets showed significant differences analyzed by Bonferroni’s post-hoc test.

## Data Availability

Data sharing not applicable.
